# The Buffer Capacity and Calcium Concentration of Water Influence the Microbial Species Diversity, Grain Growth, and Metabolite Production During Water Kefir Fermentation

**DOI:** 10.3389/fmicb.2019.02876

**Published:** 2019-12-13

**Authors:** David Laureys, Maarten Aerts, Peter Vandamme, Luc De Vuyst

**Affiliations:** ^1^Research Group of Industrial Microbiology and Food Biotechnology, Faculty of Sciences and Bioengineering Sciences, Vrije Universiteit Brussel, Brussels, Belgium; ^2^Laboratory of Microbiology, Department of Biochemistry and Microbiology, Faculty of Sciences, Ghent University, Ghent, Belgium

**Keywords:** water kefir, yeast, lactic acid bacteria, bifidobacteria, calcium, buffer

## Abstract

Eight water kefir fermentation series differing in buffer capacity and calcium concentration of the water used for fermentation were studied during eight backslopping steps. High buffer capacities resulted in high pH values and high calcium concentrations resulted in low pH values at the end of each backslopping step. When the water buffer capacity and/or calcium concentration were below certain minima, the water kefir grain growth decreased gradually over multiple backsloppings. High water buffer capacities resulted in high concentrations of residual total carbohydrate concentrations and low metabolite concentrations. Further, high water buffer capacities resulted in high ratios of lactic acid bacteria to yeasts, which was reflected in high molar ratios of the concentrations of lactic acid to ethanol and acetic acid to ethanol. The most prevalent microorganisms of the water kefir grain inoculum and grains of all fermentation series at the end of the eighth backslopping step were *Lactobacillus hilgardii*, *Lactobacillus nagelii*, *Lactobacillus paracasei*, *Bifidobacterium aquikefiri*, *Saccharomyces cerevisiae*, and *Dekkera bruxellensis*. These microbial communities were influenced by the water buffer capacity and had an impact on the substrate consumption and metabolite production during water kefir fermentation.

## Introduction

Water kefir is a traditional fermented beverage that is produced worldwide under a variety of names (Pothakos et al., 2016). The water kefir fermentation process is started by adding water kefir grains (the inoculum) to a mixture of water, (dried) fruits, and sugar; it is usually performed at room temperature under anaerobic conditions for 2–4 days (Gulitz et al., 2011, 2013; Marsh et al., 2013; Stadie et al., 2013; Laureys and De Vuyst, 2014, 2017; Laureys et al., 2017, 2018). After fermentation, the water kefir liquor is separated from the water kefir grains by sieving to obtain a slightly sweet, alcoholic, acidic, and sparkling beverage with a yellowish color and a fruity taste and aroma.

The water-insoluble, translucent, and brittle water kefir grains are composed of glucan-type exopolysaccharides (EPS) (Horisberger, 1969; Waldherr et al., 2010), and harbor the water kefir microorganisms (Waldherr et al., 2010; Gulitz et al., 2011; Laureys and De Vuyst, 2014). When the water kefir grain inoculum is added to the water kefir liquor, part of the microorganisms detach from the grains into the liquor, but the majority remains always associated with the grains (Laureys and De Vuyst, 2014). The key microorganisms of water kefir fermentation are *Lactobacillus paracasei*, *Lactobacillus hilgardii*, *Lactobacillus nagelii*, and *Saccharomyces cerevisiae* (Laureys and De Vuyst, 2017). Other species of lactic acid bacteria (LAB), yeasts, acetic acid bacteria (AAB), and/or bifidobacteria may occur too (Waldherr et al., 2010; Gulitz et al., 2011, 2013; Laureys and De Vuyst, 2014; Laureys et al., 2016, 2018). These microorganisms convert sucrose into water kefir grain EPS, ethanol, carbon dioxide, lactic acid, glycerol, acetic acid, mannitol, and a variety of aroma compounds (Laureys and De Vuyst, 2014, 2017; Laureys et al., 2018). The water kefir grain mass usually increases during fermentation, due to the production of glucan EPS from sucrose by glucansucrases (Pidoux et al., 1988, 1990; Pidoux, 1989; Waldherr et al., 2010; Laureys and De Vuyst, 2014, 2017; Laureys et al., 2018). The activity of these extracellular enzymes depends on the environmental conditions, which may thus influence the water kefir grain growth during fermentation (Waldherr et al., 2010). Low grain growth is a common problem during water kefir fermentation, and can prevent successful continuation and upscaling of a water kefir production process (Laureys et al., 2017).

Water kefir grain growth during fermentation is mainly influenced by the water kefir grain inoculum, besides environmental factors such as available nutrients, and can change gradually over the course of multiple backsloppings (Waldherr et al., 2010; Laureys and De Vuyst, 2017; Laureys et al., 2018). Nutrient availability and hence the capacity to use different fruits and other matrices, such as various vegetable and fruit juices, have been investigated for water kefir production (Zanirati et al., 2015; Corona et al., 2016; Fiorda et al., 2016a, b; Randazzo et al., 2016; Viana et al., 2017; Laureys et al., 2018).

*Lactobacillus hilgardii* is probably responsible for water kefir grain growth (Pidoux et al., 1990; Waldherr et al., 2010), but other LAB strains isolated from water kefir fermentations can also produce EPS from sucrose, as is the case for *Lb*. *nagelii*, *Leuconostoc mesenteroides*, and *Lactobacillus hordei* (Gulitz et al., 2011). However, the presence of EPS-producing *Lb*. *hilgardii* strains is not sufficient for good water kefir grain growth during fermentation. The water kefir grain growth may decrease as a result of excessive acidic stress during fermentation (Laureys and De Vuyst, 2017). Indeed, the activity of glucansucrase from *Lb*. *hilgardii* decreases from 60 at pH 3.6 to 10% at pH 3.2 (Waldherr et al., 2010), supporting that the pH during fermentation may have an effect on the water kefir grain growth. Hence, the influence of acidic stress on the water kefir grain growth and other characteristics of the water kefir fermentation process needs to be investigated in detail, for instance through the buffer capacity of the water used.

Glucansucrases have a calcium-binding region near their active center and need calcium ions for optimal activity (Yokoi and Watanabe, 1992; Kralj et al., 2004; Vujičć-Žagar et al., 2010; Leemhuis et al., 2013). This suggests that calcium may influence the water kefir grain growth during fermentation. Calcium is one of the most abundant minerals in water, but its concentration varies widely depending on the water source (Misund et al., 1999). Hence, the influence of the calcium concentration of the water used on the water kefir grain growth and other characteristics of the water kefir fermentation process needs to be investigated in detail.

This work aimed to investigate the influence of the buffer capacity and calcium concentration of the water used for fermentation on the microbial species diversity, water kefir grain growth, substrate consumption, and metabolite production during water kefir fermentation.

## Materials and Methods

### Fermentations

Starting with a water kefir grain inoculum obtained from a private person (Ghent, Belgium), pre-fermentations were carried out to obtain >1300 g of water kefir grain mass, as described before (Laureys et al., 2018). This water kefir grain mass was used as inoculum to start eight series of water kefir fermentations (Table 1), which differed in (i) the buffer capacity, indicated with the letter B preceded by a number representing the concentration of HCO_3_^–^ (added as KHCO_3_; Sigma–Aldrich, Saint Louis, MO, United States) relative to a basic concentration of 313 mg l^–1^ (=1), which corresponds with the buffer capacity of untreated tap water (Brussels, Belgium) (indications 0B, 1B, and 2B) or (ii) the calcium concentration of the water used for fermentation, indicated with the letters Ca preceded by a number representing the concentration of Ca^2+^ [added as CaCl_2_.2H_2_O (Merck, Darmstadt, Germany)] relative to a basic concentration of 50 mg l^–1^, which corresponds with an average calcium concentration in drinking water (Misund et al., 1999) (indications 0Ca, 1Ca, and 4Ca). Hereto, ultrapure water (18.2 MΩ⋅cm at 25°C) was used, obtained from a gradient A10 Milli-Q water purification system (EMD Millipore, Billerica, MA, United States), that was supplemented with either KHCO_3_ (0, 313, or 626 mg l^–1^) or CaCl_2_.2H_2_O (0, 50, or 200 mg l^–1^), corresponding with the concentrations of the fermentations mentioned above. They are further referred to as 0B0Ca and 0B1Ca; 1B0Ca, 1B1Ca, and 1B4Ca; and 2B1Ca and 2B4Ca (representing fermentations with different buffer capacities and concomitant increasing calcium concentrations) or 0B0Ca and 1B0Ca; 0B1Ca, 1B1Ca, and 2B1Ca; or 1B4Ca and 2B4Ca (representing fermentations with different calcium concentrations and concomitant increasing buffer capacity). Untreated tap water was used for a control fermentation (further referred to as TAP).

**TABLE 1:** Water kefir fermentation series, differing in the buffer capacity and calcium concentration of the water used for fermentation, examined throughout this study.

**Fermentation series**	**Buffer capacity (mg l^–1^ of HCO_3_^–^)**	**Calcium concentration (mg l^–1^ of Ca^2+^)**
0B0Ca	0	0
0B1Ca	0	50
1B0Ca	313	0
1B1Ca	313	50
1B4Ca	313	200
2B1Ca	626	50
2B4Ca	626	200

Each fermentation series was performed in independent biological triplicates; all the results are presented as the mean ± standard deviation. All fermentations were carried out in 250-ml glass recipients (Schott bottles) equipped with a water lock of polytetrafluoroethylene. They were started by adding 50 g of non-rinsed water kefir grains to 10 g of sugar (Candico Bio, Merksem, Belgium), 5 g of dried figs (King Brand, Naziili, Turkey), and 160 ml of water of the appropriate composition. The bottles were incubated in a water bath at 21°C. At the start and at the end of each backslopping step, the fermentation bottles were gently turned to mix their contents. For each fermentation bottle, the backslopping practice was applied eight times (every 3 days) by separating the water kefir grains from the water kefir liquors through sieving, after which 50 g of non-rinsed water kefir grains were recultivated in fresh medium with the same composition as before.

### pH, Water Kefir Grain Wet and Dry Mass, and Water Kefir Grain Growth Determinations

The pH, the water kefir grain wet mass, the water kefir grain growth, and the water kefir grain dry mass were determined at the end of backslopping step 8 of each fermentation series, as described before (Laureys and De Vuyst, 2014), except for the fact that the water kefir grains were not rinsed with saline. The water kefir grains were assessed visually too.

### Substrate and Metabolite Concentration Determinations

The substrate and metabolite concentrations were determined for the liquors of the eight fermentation series at the end of backslopping steps 1 and 8. Samples were prepared as described before (Laureys and De Vuyst, 2014). The concentrations of sucrose, glucose, and fructose were determined through high-performance anion exchange chromatography with pulsed amperometric detection (HPAEC-PAD), as described before (Laureys and De Vuyst, 2014), except that 100 μl of cell-free supernatant was added to 400 μl of ultrapure water, and 100 μl of this dilution was added to 900 μl of deproteinization solution (Laureys and De Vuyst, 2014). The concentrations of D- and L-lactic acid and acetic acid were determined through high-performance liquid chromatography with ultraviolet detection (HPLC–UV), those of glycerol and mannitol through HPAEC-PAD, those of ethanol through gas chromatography with flame ionization detection (GC–FID), and those of aroma compounds through static headspace gas chromatography with mass spectrometry detection (SH–GC–MS), as described before (Laureys and De Vuyst, 2014). The concentrations of the aroma compounds were compared with their threshold values as described before (Laureys and De Vuyst, 2014).

### Carbon Recovery

At the end of each backslopping step, the carbon recovery was calculated, as described before (Laureys and De Vuyst, 2014). To this end, the total amount of carbon including that of the figs added to the fermentation was taken into account. Average mono- and disaccharide contents (m m^–1^) of dried figs (48%) were used, as obtained from the National Nutrient Database for Standard Reference (release 26^1^).

### Microbial Enumerations

The viable counts of the presumptive LAB, yeasts, and AAB were determined for the non-rinsed water kefir grains of the inoculum and the eight fermentation series at the end of backslopping step 8. Those of the presumptive LAB and AAB were determined as described before (Laureys and De Vuyst, 2014), except that an additional antibiotic, amphotericin B (final concentration of 0.0025 g l^–1^; Sigma–Aldrich), was added to the de Man–Rogosa–Sharpe (MRS) and modified deoxycholate–mannitol–sorbitol (mDMS) agar media. Yeast extract–peptone–dextrose (YPD) agar medium, supplemented with chloramphenicol (final concentration of 0.1 g l^–1^; Sigma–Aldrich), was used to determine the viable counts of the presumptive yeasts, as described before (Laureys and De Vuyst, 2014).

### Culture-Dependent Microbial Species Diversity Analyses

The culture-dependent microbial species diversity of the LAB and yeasts were determined for the non-rinsed water kefir grains of the inoculum and the eight fermentation series at the end of backslopping step 8 by randomly picking 10–20% of the total number of colonies from the respective agar media with 30–300 colonies. Each isolate was sub-cultivated on its respective agar medium until the third generation, which was stored at −80°C in YPD medium supplemented with 25% (v v^–1^) of glycerol as cryoprotectant, and used for dereplication via matrix-assisted laser desorption/ionization time-of-flight mass spectrometry (MALDI–TOF MS) fingerprinting, as described before (Spitaels et al., 2014). The fingerprint peptide patterns, ranging from 2 to 20 kDa, were clustered numerically into similarity trees using the Pearson correlation coefficient (PCC) and the unweighted pair group method with arithmetic mean (UPGMA) algorithm by means of the Bionumerics software version 5.10 (Applied Maths, Sint-Martens-Latem, Belgium). Representative bacterial and yeast isolates within each cluster were identified by sequencing part of their genomic DNA [16S rRNA gene in the case of bacteria and 26S large subunit (LSU) rRNA gene and internal transcribed spacer (ITS) region in the case of yeasts], as described before (Laureys and De Vuyst, 2014).

### Exopolysaccharide Production Capacity

The EPS production capacity was assessed visually after growing all bacterial isolates on MRS agar medium supplemented with 10 g l^–1^ of sucrose at 30°C for 7 days.

### Culture-Independent Microbial Species Diversity Analyses

The culture-independent microbial species diversity of bacteria and yeasts was determined for the water kefir liquors and the non-rinsed water kefir grains of the inoculum and the eight fermentation series at the end of backslopping step 8 after preparing total DNA extracts from the cell pellets of the water kefir liquors or 0.2 g of crushed water kefir grains, respectively. Cell pellets of the water kefir liquors were obtained after centrifugation (7,200 × *g*, 20 min, 4°C) of 40 ml of water kefir liquors and discarding the supernatants. Total DNA extraction was performed by means of an optimized protocol, including enzymatic, mechanical, and chemical cell lysis, phenol/chloroform/isoamyl alcohol extraction and precipitation, and column purification, as described previously (Laureys et al., 2018).

Amplification of selected genomic fragments in the total DNA with the universal prokaryotic primer pair (V3), the LAB-specific primer pair (LAC), the *Bifidobacterium*-specific primer pair (Bif), and the universal eukaryotic primer pair (Yeast), followed by denaturing gradient gel electrophoresis (DGGE) of these PCR amplicons, yielded culture-independent microbial community gel profiles, from which selected bands were cut that were identified through sequencing, as described before (Laureys and De Vuyst, 2014).

### Statistics

Differences between the eight water kefir fermentation series were tested by ANOVA, followed by Fisher’s least significant difference (LSD) tests for a series of *post hoc* pairwise comparisons (de Winter, 2013). Two-tailed Spearman correlation coefficients between test variables were calculated for all fermentation series with defined buffer capacities and calcium concentrations of the water, excluding the control fermentation with tap water.

The correlation coefficients between the buffer capacity of the water and the characteristics of the water kefir fermentation processes were always controlled for the calcium concentration of the water, and those between the calcium concentration of the water and the characteristics of the water kefir fermentation processes were always controlled for the buffer capacity. The correlation coefficients between different characteristics of the water kefir fermentation processes were not controlled.

All statistical tests were performed in R 3.2.0 (R Core Team, 2013) with a significance level of 0.05.

## Results

### pH, Water Kefir Grain Wet and Dry Mass, and Water Kefir Grain Growth

For the eight fermentation series examined, at the end of backslopping step 1, high water buffer capacities resulted in high pH values and high calcium concentrations resulted in low pH values (Supplementary Table S1). Indeed, the pH at the end of backslopping step 1 correlated positively with the buffer capacity (controlled for the calcium concentration) and negatively with the calcium concentration (controlled for the buffer capacity) (Table 2). The water buffer capacity and calcium concentration had no significant influence on the water kefir grain growth, which was approximately 58% for all fermentation series (Supplementary Table S1).

**TABLE 2:** Spearman correlation coefficients (SCCs) between the buffer capacity (controlled for the calcium concentration) or the calcium concentration (controlled for the buffer capacity) of the water used for fermentation and the characteristics of the eight water kefir fermentation series examined (differing in the buffer capacity and calcium concentration of the water used for fermentation), at the end of backslopping steps 1 and 8.

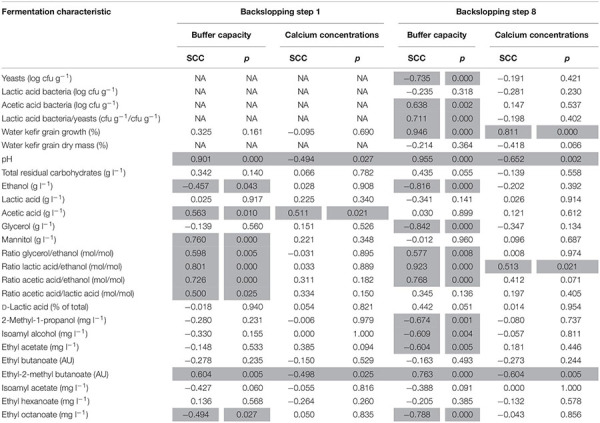

*Significant correlations have a gray background. NA, not available; AU, arbitrary units.*

**FIGURE 1:**
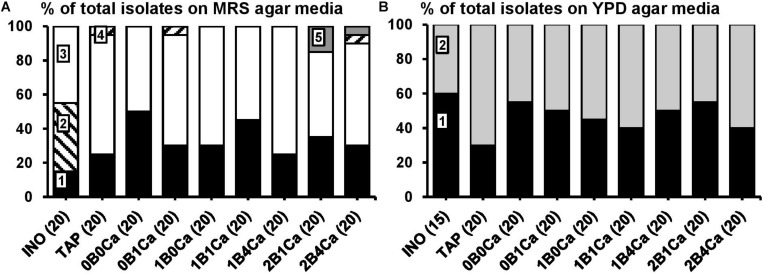
Culture-dependent species diversity on the water kefir grains of the inoculum (INO) and eight water kefir fermentation series differing in the buffer capacity and calcium concentrations of the water used for fermentation at the end of backslopping step 8. The closest known type strains of the sequenced fragments are given. **(A)** Isolates from MRS agar media: 1, *Lactobacillus paracasei* (99% identity; GenBank accession no. AP012541); 2, *Lactobacillus hilgardii* (100% identity; accession no. LC064898); 3, *Lactobacillus nagelii* (99% identity; accession no. NR112754); 4, *Lactobacillus harbinensis* (100% identity; accession no. NR113969); and 5, *Leuconostoc pseudomesenteroides* (99% identity; accession no. LC096220). **(B)** Isolates from YPD agar media: 1, *Saccharomyces cerevisiae* [LSU (99% identity; accession no. KC881066) and ITS (99% identity; accession no. KC881067)]; and 2, *Dekkera bruxellensis* [LSU (99% identity; accession no. AY969049) and ITS (99% identity; accession no. NR111030)]. LSU, large subunit rRNA gene; ITS, internal transcribed spacer.

Over the course of the eight backslopping steps in all fermentation series, the pH values of the eight fermentation series decreased slightly, and this was more pronounced for the fermentation series with a large decrease of the water kefir grain growth (Figure 1 and Supplementary Tables S2, S3). When the water buffer capacity and/or calcium concentration were below certain minima, the water kefir grain growth decreased significantly already at the end of backslopping step 2. This decrease continued gradually over the course of the eight backslopping steps (Figure 1). The minimum buffer capacity and calcium concentration to obtain a water kefir grain growth similar to that of the control fermentation series with tap water at the end of backslopping step 8 were 313 mg l^–1^ of HCO_3_^–^ and 200 mg l^–1^ of Ca^2+^ (fermentation series 1B4Ca), or 626 mg l^–1^ of HCO_3_^–^ and 50 mg l^–1^ of Ca^2+^ (2B1Ca). A water buffer capacity and/or calcium concentration above these minima did not further increase the water kefir grain growth.

The results at the end of backslopping step 8 were in line with those at the end of backslopping step 1 in all fermentation series, whereby high water buffer capacities resulted in high pH values and high calcium concentrations resulted in low pH values (Table 3). Indeed, the pH at the end of backslopping step 8 correlated again positively with the buffer capacity of the water (controlled for the calcium concentration) and negatively with the calcium concentration of the water (controlled for the buffer capacity) (Table 2). The water kefir grain growth at the end of backslopping step 8 ranged from 2.7 ± 0.5 for the fermentation series 0B0Ca to 52.0 ± 2.3% for the fermentation series 2B4Ca (Table 3), and correlated positively with the buffer capacity and the calcium concentration of the water (Table 2) and the pH (0.801; *p* < 0.001).

**TABLE 3:** Characteristics of eight water kefir fermentation series differing in the buffer capacity and calcium concentration of the water used for fermentation at the end of backslopping step 8 [control fermentation with tap water (TAP); fermentations with different buffer capacity, and concomitant increasing calcium concentrations (0B0Ca and 0B1Ca; 1B0Ca, 1B1Ca, and 1B4Ca; and 2B1Ca and 2B4Ca); and fermentations with different calcium concentrations and concomitant increasing buffer capacity (0B0Ca and 1B0Ca; 0B1Ca, 1B1Ca, and 2B1Ca; and 1B4Ca and 2B4Ca)].

**Characteristic**	**TAP**	**0B0Ca**	**0B1Ca**	**1B0Ca**	**1B1Ca**	**1B4Ca**	**2B1Ca**	**2B4Ca**
Yeasts (log cfu g^–1^)	7.5 ± 0.1^bc^	7.7 ± 0.1^a^	7.7 ± 0.1^ab^	7.5 ± 0.1^c^	7.4 ± 0.1^c^	7.3 ± 0.1^cd^	7.2 ± 0.1^d^	7.4 ± 0.2^cd^
Lactic acid bacteria (log cfu g^–1^)	8.4 ± 0.1	8.6 ± 0.1	8.5 ± 0.1	8.4 ± 0.2	8.3 ± 0.1	8.3 ± 0.1	8.4 ± 0.1	8.4 ± 0.1
Acetic acid bacteria (log cfu g^–1^)	4.7 ± 0.3^a^	3.5 ± 0.4^d^	3.7 ± 0.5^cd^	3.9 ± 0.6^bd^	4.2 ± 0.3^abc^	4.4 ± 0.2^ab^	4.8 ± 0.1^a^	4.4 ± 0.5^abc^
Lactic acid bacteria/yeasts (cfu/cfu)	8.9 ± 0.8^bc^	7.7 ± 2.3^c^	7.2 ± 0.8^c^	9.8 ± 2.8^bc^	9.0 ± 3.1^bc^	9.1 ± 2.3^bc^	14.8 ± 2.1^a^	12.1 ± 3.3^ab^
Water kefir grain growth (%)	47.9 ± 0.7^a^	2.7 ± 0.5^d^	5.4 ± 0.6^d^	17.5 ± 1.8^c^	31.2 ± 8.7^b^	47.2 ± 0.5^a^	50.9 ± 2.9^a^	52.0 ± 2.3^a^
Water kefir grain dry mass (%)	14.1 ± 0.3^bc^	14.2 ± 0.3^bc^	14.4 ± 0.5^ac^	14.6 ± 0.2^ab^	15.0 ± 0.4^a^	13.9 ± 0.4^c^	14.0 ± 0.3^bc^	13.0 ± 0.6^d^
pH	3.45 ± 0.01^bc^	3.17 ± 0.01^e^	3.14 ± 0.02^e^	3.41 ± 0.04^c^	3.43 ± 0.10^c^	3.32 ± 0.03^d^	3.60 ± 0.01^a^	3.52 ± 0.02^b^
Sucrose (g l^–1^)	1.3 ± 0.1^bc^	1.0 ± 0.3^c^	2.6 ± 1.7^ab^	3.9 ± 1.4^a^	2.1 ± 0.5^bc^	1.4 ± 0.2^bc^	1.3 ± 0.2^bc^	1.5 ± 0.1^bc^
Glucose (g l^–1^)	0.7 ± 0.6	0.2 ± 0.1	0.6 ± 0.7	1.2 ± 0.4	1.8 ± 1.4	0.3 ± 0.4	0.7 ± 0.3	0.1 ± 0.1
Fructose (g l^–1^)	10.1 ± 4.0^a^	2.7 ± 1.0^b^	3.7 ± 2.8^b^	6.7 ± 1.9^ab^	10.8 ± 5.1^a^	7.5 ± 4.7^ab^	10.6 ± 2.1^a^	7.5 ± 0.3^ab^
Total residual carbohydrates (g l^–1^)	12.1 ± 4.7	3.9 ± 1.4	7.0 ± 5.2	11.8 ± 0.9	14.6 ± 7.1	9.2 ± 5.3	12.6 ± 2.7	9.1 ± 0.3
Ethanol (g l^–1^)	17.7 ± 2.2^cd^	31.6 ± 0.4^a^	29.5 ± 2.5^a^	22.8 ± 0.6^b^	18.8 ± 5.0^bc^	18.7 ± 2.5^c^	14.5 ± 0.2^d^	17.1 ± 0.6^cd^
Lactic acid (g l^–1^)	2.63 ± 0.38^d^	3.40 ± 0.12^a^	3.30 ± 0.29^ab^	2.92 ± 0.25^ad^	2.73 ± 0.41^cd^	2.91 ± 0.15^ad^	2.83 ± 0.33^d^	3.2 ± 0.22^cd^
Acetic acid (g l^–1^)	1.05 ± 0.10	1.26 ± 0.03	1.21 ± 0.08	1.00 ± 0.08	1.05 ± 0.20	1.14 ± 0.10	1.25 ± 0.16	1.24 ± 0.07
Glycerol (g l^–1^)	1.87 ± 0.27^cd^	2.76 ± 0.10^a^	2.50 ± 0.14^b^	2.01 ± 0.07^c^	1.84 ± 0.21^cd^	1.82 ± 0.06^cd^	1.67 ± 0.07^d^	1.74 ± 0.02^d^
Mannitol (g l^–1^)	0.59 ± 0.04^bc^	0.74 ± 0.18^b^	0.68 ± 0.06^b^	0.43 ± 0.15^c^	0.56 ± 0.11^bc^	0.67 ± 0.16^bc^	1.00 ± 0.22^a^	0.58 ± 0.12^bc^
Glycerol/ethanol (mmol/mol)	53 ± 3^ab^	44 ± 2^cd^	42 ± 2^d^	44 ± 3^cd^	50 ± 7^bc^	49 ± 7^bcd^	58 ± 2^a^	51 ± 2^ac^
Lactic acid/ethanol (mmol/mol)	76 ± 5^bc^	55 ± 1^d^	57 ± 1^d^	65 ± 6^cd^	75 ± 10^bc^	80 ± 7^b^	100 ± 11^a^	95 ± 4^a^
Acetic acid/ethanol (mmol/mol)	45 ± 3^bc^	30 ± 1^e^	32 ± 1^e^	34 ± 4^de^	44 ± 12^cd^	48 ± 10^bc^	66 ± 8^a^	55 ± 2^ab^
Acetic acid/lactic acid (mol/mol)	0.60 ± 0.03	0.56 ± 0.03	0.55 ± 0.02	0.51 ± 0.05	0.58 ± 0.09	0.59 ± 0.08	0.66 ± 0.04	0.58 ± 0.2
D-Lactic acid (% of total)	46.2 ± 0.2	45.6 ± 0.1	45.5 ± 1.0	46.6 ± 0.7	46.5 ± 0.8	46.3 ± 0.6	46.4 ± 1.3	46.9 ± 0.8
Carbon recovery (%)	99.7 ± 1.1^b^	105.2 ± 0.7^a^	104.4 ± 0.6^a^	99.7 ± 1.5^b^	99.3 ± 0.8^b^	99.2 ± 0.2^b^	95.7 ± 2.7^c^	96.9 ± 1.1^c^
2-Methyl-1-propanol (mg l^–1^)	8.7 ± 2.0^c^	13.0 ± 0.7^ab^	13.7 ± 3.9^a^	10.7 ± 0.3^ac^	9.3 ± 2.9^c^	9.9 ± 1.7^bc^	8.3 ± 0.3^c^	9.4 ± 1.1^c^
Isoamyl alcohol (mg l^–1^)	40.0 ± 4.1^cd^	50.1 ± 1.3^ab^	51.2 ± 8.0^a^	48.4 ± 1.7^ac^	40.0 ± 8.4^cd^	44.6 ± 7.5^ad^	36.5 ± 4.4^d^	40.8 ± 1.6^bcd^
Ethyl acetate (mg l^–1^)	13.1 ± 0.9^c^	19.4 ± 1.6^ab^	23.6 ± 8.2^a^	12.9 ± 1.7^c^	13.3 ± 1.3^c^	13.6 ± 3.4^bc^	12.7 ± 1.0^c^	14.9 ± 1.8^bc^
Isoamyl acetate (mg l^–1^)	0.11 ± 0.02	0.15 ± 0.02	0.17 ± 0.04	0.15 ± 0.01	0.13 ± 0.04	0.14 ± 0.02	0.13 ± 0.01	0.14 ± 0.01
Ethyl hexanoate (mg l^–1^)	0.29 ± 0.05	0.38 ± 0.13	0.37 ± 0.07	0.43 ± 0.02	0.40 ± 0.14	0.33 ± 0.05	0.31 ± 0.01	0.34 ± 0.03
Ethyl octanoate (mg l^–1^)	0.33 ± 0.06^de^	0.58 ± 0.01^ab^	0.69 ± 0.19^a^	0.49 ± 0.10^bc^	0.35 ± 0.11^cde^	0.43 ± 0.03^bd^	0.27 ± 0.04^e^	0.32 ± 0.03^de^

*0B0Ca, no HCO_3_^–^ and Ca^*2*+^; 0B1Ca, no HCO_3_^–^ and 50 mg l^–1^ of Ca^*2*+^; 1B0Ca, 313 mg l^–1^ of HCO_3_^–^ and no Ca^*2*+^; 1B1Ca, 313 mg l^–1^ of HCO_3_^–^ and 50 mg l^–1^ of Ca^*2*+^; 1B4Ca, 313 mg l^–1^ of HCO_3_^–^ and 200 mg l^–1^ of Ca^*2*+^; 2B1Ca, 626 mg l^–1^ of HCO_3_^–^ and 50 mg l^–1^ of Ca^*2*+^; and 2B4Ca, 626 mg l^–1^ of HCO_3_^–^ and 200 mg l^–1^ of Ca^*2*+^. Significant differences between the series are indicated with different superscripts (a–e).*

The water kefir grain dry mass at the end of backslopping step 8 was approximately 14% (m m^–1^) for all fermentation series (Table 3). Visual assessment of the water kefir grains at the end of backslopping step 8 indicated that they were smaller when the water kefir grain growth was lower.

### Substrate Consumption and Metabolite Production

The total residual carbohydrate concentrations in all fermentation series were 5.3–10.9 g l^–1^ at the end of backslopping step 1 (Supplementary Table S1), and 3.9–14.6 g l^–1^ at the end of backslopping step 8 (Table 3), whereby fructose was always the main residual carbohydrate. Although the concentrations of the total residual carbohydrates did not differ significantly between the fermentation series at the end of backslopping steps 1 and 8, they were always lowest in the fermentation series with the lowest water buffer capacities (0B0Ca and 0B1Ca) and always highest in the fermentation series with the highest water buffer capacities (TAP, 2B1Ca, and 2B4Ca). The total residual carbohydrate concentrations correlated positively with the pH at the end of backslopping steps 1 (0.597; *p* = 0.005) and 8 (0.491; *p* = 0.025).

The fermentation series with the lowest water buffer capacity and calcium concentration (0B0Ca) resulted in the highest concentrations of ethanol at the end of backslopping step 1 (Supplementary Table S1), and the highest concentrations of ethanol, lactic acid, and glycerol at the end of backslopping step 8 (Table 3). Indeed, the buffer capacity of the water correlated negatively with the concentrations of ethanol and positively with the concentrations of acetic acid and mannitol at the end of backslopping step 1 (Table 2). The buffer capacity of the water correlated negatively with the concentrations of ethanol and glycerol at the end of backslopping step 8 (Table 2). Further, the buffer capacity of the water correlated positively with the ratios of the concentrations of glycerol to ethanol, lactic acid to ethanol, acetic acid to ethanol, and acetic acid to lactic acid at the end of backslopping steps 1 and 8. At the end of backslopping steps 1 and 8, the buffer capacity of the water correlated positively with the concentrations of ethyl-2-methyl butanoate and negatively with the concentrations of ethyl decanoate, whereas the calcium concentration of the water correlated negatively with the concentrations of ethyl-2-methyl butanoate. The calcium concentration of the water correlated positively with the concentration of acetic acid at the end of backslopping step 1 and the ratios of the concentrations of lactic acid to ethanol at the end of backslopping step 8.

At the end of backslopping step 1, the concentrations of ethanol in the different fermentation series correlated positively with the concentrations of glycerol (0.662; *p* < 0.001) and total lactic acid (0.588; *p* = 0.006), but not with those of acetic acid (−0.114; *p* = 0.613). At the end of backslopping step 8, the concentrations of ethanol correlated positively with the concentrations of glycerol (0.932; *p* < 0.001) and lactic acid (0.645; *p* = 0.002), but not with those of acetic acid (−0.032; *p* = 0.890).

At the end of backslopping step 1, the concentrations of ethanol in the different fermentation series correlated positively with the concentrations of ethyl butanoate (0.895; *p* < 0.001), 2-methyl-1-propanol (0.753; *p* < 0.001), isoamyl alcohol (0.736; *p* < 0.001), isoamyl acetate (0.945; *p* < 0.001), ethyl hexanoate (0.658; *p* = 0.002), and ethyl octanoate (0.736; *p* < 0.001), but not with the concentrations of ethyl acetate (0.377; *p* = 0.093) and ethyl-2-methylbutanoate (0.143; *p* = 0.535). At the end of backslopping step 8 in the different fermentation series, the concentrations of ethanol correlated positively with the concentrations of ethyl acetate (0.677; *p* < 0.001), ethyl butanoate (0.561; *p* = 0.009), 2-methyl-1-propanol (0.879; *p* < 0.001), isoamyl alcohol (0.848; *p* < 0.001), isoamyl acetate (0.648; *p* = 0.002), ethyl hexanoate (0.547; *p* = 0.011), and ethyl octanoate (0.857; *p* < 0.001), and negatively with the concentrations of ethyl-2-methylbutanoate (−0.536; *p* = 0.013). The threshold values of isoamyl alcohol, ethyl acetate, isoamyl acetate, ethyl hexanoate, and ethyl octanoate were exceeded in all fermentations at the end of backslopping steps 1 and 8.

At the end of backslopping step 1, the concentrations of total lactic acid in the different fermentation series correlated positively with the concentrations of acetic acid (0.534; *p* < 0.014), but not with the pH (−0.162; *p* = 0.480). At the end of backslopping step 8, the concentrations of total lactic acid in the different fermentation series correlated positively with the concentrations of acetic acid (0.532; *p* = 0.014) and negatively with the pH (−0.436; *p* = 0.049).

At the end of backslopping step 1, the concentrations of acetic acid in the different fermentation series correlated positively with the pH (0.514; *p* = 0.018) and the concentrations of mannitol (0.486; *p* = 0.027), but not with the concentrations of glycerol (0.143; *p* = 0.535). At the end of backslopping step 8, the concentrations of acetic acid in the different fermentation series correlated positively with the concentrations of mannitol (0.564; *p* = 0.009), but not with the pH (−0.073; *p* = 0.754) or the concentrations of glycerol (0.027; *p* = 0.908). The concentrations of glycerol and mannitol did not correlate at the end of backslopping steps 1 (−0.096; *p* = 0.678) and 8 (−0.106; *p* = 0.645).

Overall, a carbon recovery of approximately 100% was found in all fermentation series at the end of backslopping steps 1 (Supplementary Table S1) and 8 (Table 3), but the carbon recovery correlated negatively with the water kefir grain growth at the end of backslopping step 8 (−0.890; *p* < 0.001).

### Microbial Enumerations

The buffer capacity of the water in the different fermentation series did not correlate with the viable counts of the LAB on the water kefir grains, correlated negatively with those of the yeasts, and positively with those of the AAB (Table 2). This resulted in a positive correlation between the water buffer capacity and the ratios of the viable counts of the LAB to the yeasts on the water kefir grains. The calcium concentration had no significant influence on the viable counts of the microorganisms on the water kefir grains.

The water kefir grain growth in the different fermentation series correlated negatively with the viable counts of the yeasts (−0.797; *p* < 0.001) and LAB (−0.528; *p* = 0.014) on the water kefir grains, and positively with those of the AAB (0.690; *p* = 0.001). Further, the water kefir grain growth correlated positively with the ratios of the viable counts of the LAB to the yeasts (0.592; *p* = 0.005) on the water kefir grains. The total residual carbohydrate concentrations correlated negatively with the viable counts of the yeasts (−0.578, *p* = 0.007) and LAB (−0.670, *p* = 0.001), and positively with those of the AAB (0.578, *p* = 0.007) on the water kefir grains.

The viable counts of the yeasts on the water kefir grains in the different fermentation series correlated positively with the concentrations of ethanol (0.845, *p* < 0.001), but not with those of acetic acid (0.123, *p* = 0.593). The viable counts of the LAB on the water kefir grains correlated positively with the concentrations of total lactic acid (0.821, *p* < 0.001), but not with those of acetic acid (0.335, *p* = 0.138). The viable counts of the AAB on the water kefir grains did not correlate with the concentrations of acetic acid (0.132, *p* = 0.566) either. The ratios of the viable counts of the LAB to the yeasts on the water kefir grains ranged from 7 to 14 (Table 3), and correlated positively with the ratios of the concentrations of lactic acid to ethanol (0.690; *p* = 0.001) and acetic acid to ethanol (0.483; *p* = 0.028), but not with those of the concentrations of acetic acid to lactic acid (0.158; *p* = 0.491).

### Culture-Dependent Microbial Species Diversity and Exopolysaccharide Production Capacity

**FIGURE 2:**
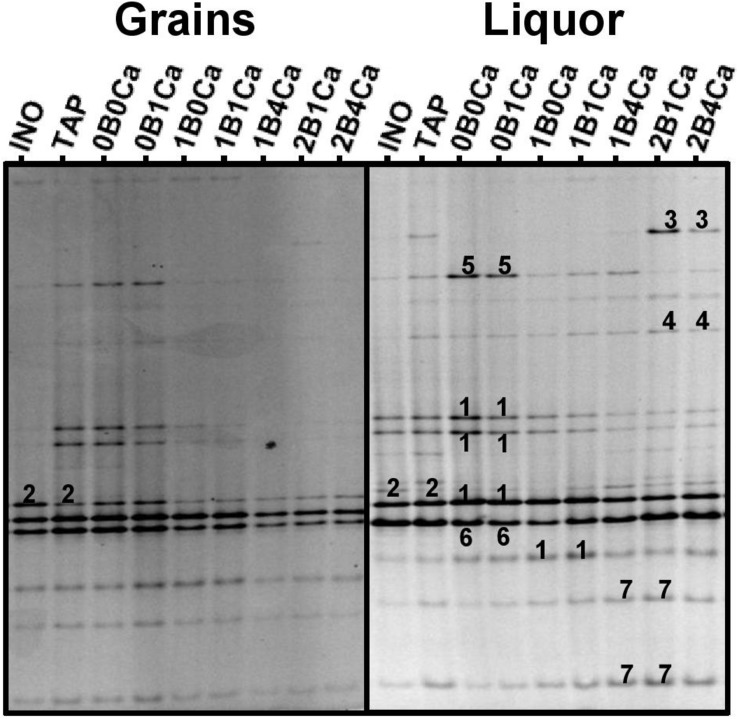
Community profiles obtained with the V3 primer pair for the water kefir grains **(left)** and water kefir liquors **(right)** of the inoculum (INO) and eight water kefir fermentation series differing in the buffer capacity and calcium concentration of the water used for fermentation at the end of backslopping step 8. The numbers indicate the bands that were sequenced and the closest known type strains of the sequenced fragments are given. With the V3 primer pair: 1, *Lactobacillus nagelii*/*ghanensis* (99% identity for both species; GenBank accession no. NR112754/NR043896); 2, *Lactobacillus hilgardii*/*diolivorans* (100% identity; accession nos. LC064898/NR037004); 3, *Leuconostoc pseudomesenteroides* (99% identity; accession no. LC096220); 4, *Lactobacillus mali/hordei* (100% identity; accession nos. NR112691/NR044394); 5, *Oenococcus kitaharae* (97% identity; accession no. NR041312); 6, *Bifidobacterium aquikefiri* (100% identity; accession no. LN849254); and 7, *Lactobacillus paracasei/casei/zeae/rhamnosus* (99% identity; accession nos. AP012541/AP012544/NR037122/JQ58098).

The main LAB species found culture-dependently in the water kefir grain inoculum were *Lb. paracasei*, *Lb. higardii*, and *Lb. nagelii* (Figure 2). At the end of backslopping step 8, *Lb*. *paracasei* and *Lb*. *nagelii* remained the main LAB species in all fermentation series, whereas *Lb. hilgardii* was not found anymore. Additionally, at the end of backslopping step 8, *Lb. harbinensis* was found in fermentation series TAP, 0B1Ca, and 2B4Ca, and *Leuconostoc pseudomesenteroides* was found in fermentation series 2B1Ca and 2B4Ca. EPS production was found for all the *Leuc*. *pseudomesenteroides* strains and for 63% of the *Lb*. *hilgardii* strains.

The main yeast species found culture-dependently in the water kefir grain inoculum were *S. cerevisiae* and *Dekkera bruxellensis*. They remained the main yeast species until the end of backslopping step 8 in all fermentation series (Figure 2).

### Culture-Independent Microbial Species Diversity

At the end of backslopping step 8, the rRNA–PCR–DGGE community profiles obtained with the four different primer pairs (V3, LAC, Bif, and Yeast) for the three independent biological replicates performed for each fermentation series were similar (data not shown).

**FIGURE 3:**
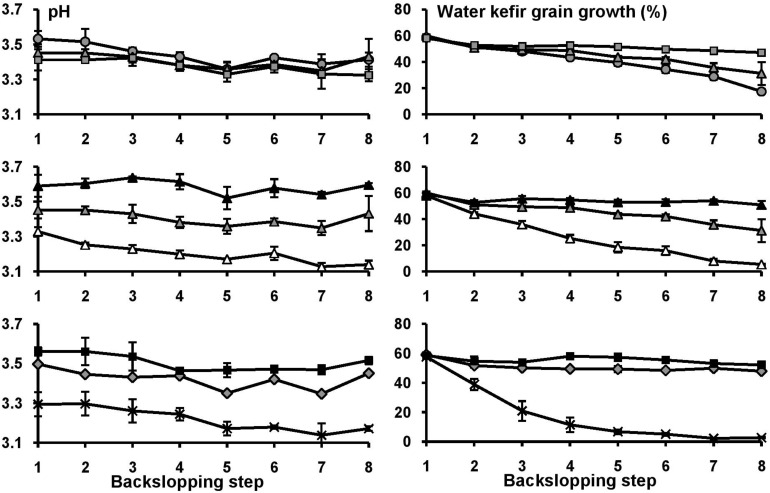
The pH and water kefir grain growth at the end of each backslopping step for eight water kefir fermentation series differing in the buffer capacity and calcium concentrations of the water used for fermentation: increasing calcium concentrations [1B0Ca (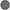), 1B1Ca (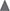), and 1B4Ca (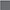)] **(top)**; increasing buffer capacity [0B1Ca (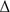), 1B1Ca (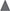), and 2B1Ca (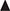)] **(middle)**; and high buffer capacity and calcium concentration [2B4Ca (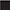)], low buffer capacity and calcium concentration [0B0Ca (X)], and tap water [TAP (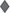)] **(bottom)**. For differences of significance, see Supplementary Tables S2, S3.

The main bands in the community profiles obtained with the V3 primer pair for the water kefir liquors and grains of the inoculum and at the end of backslopping step 8 in all fermentation series were attributed to *Lb*. *hilgardii*, *Lb*. *mali/hordei*, *Lb*. *nagelii*, *Lb*. *paracasei*, *Leuc*. *pseudomesenteroides*, *Bifidobacterium aquikefiri*, and a non-identified *Oencoccus* species (closely related to *Oenococcus kitaharae*), the latter in particular in fermentation series TAP, 0B0Ca, 0B1Ca (Figure 3). The partial 16S rRNA gene sequence of the non-identified *Oenococcus* species (213 bp) was deposited in the NCBI nucleotide database (GenBank accession no. LT220205). The relative intensities of the bands attributed to *Lb*. *nagelii*, *Lb*. *mali*/*hordei*, *Leuc*. *pseudomesenteroides*, and the non-identified *Oenococcus* species were higher for the water kefir liquors than for the water kefir grains, whereas those attributed to *Lb*. *hilgardii* were higher for the grains than for the liquors. When the water buffer capacity increased, the relative intensities of the bands attributed to *Leuc*. *pseudomesenteroides* and *Lb*. *paracasei* increased, but those of the bands attributed to *Lb*. *hilgardii* and *Lb*. *nagelii* decreased. The relative intensities of the bands attributed to *Lb*. *mali*/*hordei* were always low and those attributed to *B*. *aquikefiri* were always high for the water kefir liquors and grains of the inoculum and at the end of backslopping step 8 in all fermentation series. The community profiles obtained with the LAC primer pair confirmed the results for the LAB species obtained with the V3 primer pair (data not shown). The more or less stable presence of bands attributed to *B. aquikefiri* was confirmed by the community profiles obtained with the Bif primer pair (100% identity; accession no. LN849254) (data not shown).

The main bands in the community profiles obtained with the Yeast primer pair for the water kefir liquors and grains of the inoculum and at the end of backslopping step 8 in all fermentation series were attributed to *S. cerevisiae* (100% identity; accession no. KC881066) and *D. bruxellensis* (100% identity; accession no. AY969049) (data not shown). In the case of the liquors, the relative intensities of the bands attributed to these two yeast species were similar. In the case of the grains, the relative intensities of the bands attributed to *S*. *cerevisiae* were always higher than those of the bands attributed to *D. bruxellensis* (data not shown).

## Discussion

Water kefir fermentation depends on a characteristic consortium of microorganisms that originate from the water kefir grains, whose growth depends on many different factors (Waldherr et al., 2010; Laureys and De Vuyst, 2014, 2017; Laureys et al., 2018). The present study revealed that the buffer capacity and the calcium concentration of the water used for water kefir fermentation had an impact on the water kefir grain growth, microbial species diversity, and metabolite production during a water kefir fermentation process. A high buffer capacity and a high calcium concentration of the water used resulted in high and low pH values at the end of the fermentations, respectively. When the water buffer capacity and/or calcium concentrations were below certain minima, the water kefir grain growth decreased gradually over multiple backsloppings. Excessive acidic stress decreased the water kefir grain growth during fermentation. This decrease could not be attributed to the disappearance of the EPS-producing *Lb. hilgardii*, as this LAB species was also present when the water kefir grain growth was low. Glucansucrases produced by LAB, which are responsible for the water kefir grain growth, are extracellular enzymes, whose activity is optimal at pH 4.0–5.5 and decreases toward lower pH values (Waldherr et al., 2010; Côté and Skory, 2012). Similarly, EPS production by *Lactobacillus delbrueckii* subsp. *bulgaricus* and kefiran production by *Lactobacillus kefiranofaciens* is optimal around pH 4.5–5.5 (Kimmel et al., 1998; Cheirsilp et al., 2001). However, the water kefir grain growth remained high during the first two backslopping steps of the fermentation series without added buffer, despite their immediate low pH values. This indicated that it was more likely that low pH values compromised the water kefir grain growth by inhibiting the production of glucansucrases during fermentation than by inhibiting the glucansucrase activity itself.

The present study also revealed that an insufficient calcium concentration of the water can cause a decrease of the water kefir grain growth during fermentation. The supply of approximately 51 mg l^–1^ of calcium by adding dried figs was not sufficient to sustain good water kefir grain growth. A large part of this calcium was probably not available for the water kefir microorganisms and their enzymes. The calcium concentration of the water necessary for good water kefir grain growth depended on the buffer capacity of the water, as a higher calcium concentration was required at a lower buffer capacity. Further, a higher calcium concentration of the water resulted in a lower pH value, which was associated with lower water kefir grain growth. This indicated that the higher water kefir grain growth at higher calcium concentrations was not mediated by the pH. A high calcium concentration indeed increases the activity of reuteransucrase GTFA-ΔN from *Lactobacillus reuteri* (Kralj et al., 2004), glucansucrase GTF180-ΔN from *Lb. reuteri* (Vujičć-Žagar et al., 2010), and dextransucrase from *Leuc. mesenteroides* (Lopez and Monsan, 1980), and increases the production of kefiran by a *Lactobacillus* sp. from milk kefir grains (Yokoi and Watanabe, 1992).

Further, a high water buffer capacity seemed to be advantageous for the growth and metabolism of the LAB compared to the yeasts and resulted in high ratios of LAB to yeasts on the grains, which were reflected in high ratios of the concentrations of lactic acid to ethanol. A high water buffer capacity also resulted in high ratios of glycerol to ethanol, and high ratios of acetic acid to lactic acid. Indeed, yeasts grow optimally under acidic conditions, whereas their glycerol production is optimal around pH 6.0 (Yalcin and Ozbas, 2008).

Low water kefir grain growth was associated with small water kefir grains, high viable counts on the water kefir grains, low total residual carbohydrate concentrations, and high metabolite concentrations, confirming previous results (Laureys and De Vuyst, 2017). When the water kefir grain growth is low, the water kefir grains become small, as they are brittle and break easily during sieving and handling. This increases the viable counts of the microorganisms on the water kefir grains, as they reside mostly on their surface, resulting in a fast fermentation (Moinas et al., 1980; Neve and Heller, 2002). Additionally, low water kefir grain growth leaves more glucose available for metabolite production, further resulting in high metabolite concentrations, confirming previous results (Laureys and De Vuyst, 2017).

The identification of the microorganisms of the present study was performed with MALDI–TOF MS, a culture-dependent dereplication and identification technique for microbial isolates. That is of increasing importance in food microbiology studies, including water kefir (Viana et al., 2017; De Roos and De Vuyst, 2018; Laureys et al., 2018). *Lactobacillus hilgardii*, *Lb. nagelii*, *Lb. paracasei*, and *S. cerevisiae* were present both in the inoculum and at the end of all fermentation series, confirming their key role during water kefir fermentation (Laureys and De Vuyst, 2017). Also, *Leuc. pseudomesenteroides*, *Lb*. *harbinensis*, *Lb*. *mali/hordei*, *B. aquikefiri*, *D. bruxellensis*, and a non-identified *Oenococcus* species have been found in water kefir before (Gulitz et al., 2011, 2013; Laureys and De Vuyst, 2014; Laureys et al., 2016). The presence of *Lb. hilgardii* strains was not sufficient for good water kefir grain growth, confirming previous results (Laureys and De Vuyst, 2017). Further, *Leuc. pseudomesenteroides* was only present when the buffer capacity was high, which is consistent with its low acid tolerance compared to other LAB species (Axelsson, 2004; Ludwig et al., 2009). This microorganism also produced EPS from sucrose, but probably did not play a role in water kefir gain growth, as it was not always present, preferred the water kefir liquor over the water kefir grains, and did not influence the water kefir grain growth when it was present. This LAB species produces mainly D-lactic acid (Ludwig et al., 2009), and the proportions of D-lactic acid were indeed higher when the buffer capacity of the water was higher. The *Oenococcus* species found might represent a novel species, as its partial 16S rRNA gene sequence was only 97% identical to that of the closest known *Oenococcus* type strains, in particular *O. kitaharae* (Mattarelli et al., 2014), data that were also found via shotgun metagenomics of a water kefir microbial ecosystem (Verce et al., 2019). Its relative abundance was high at low pH values, which was conformed with the acidophilic nature of this LAB genus that occurs naturally in wine, cider, and related habitats (Ludwig et al., 2009).

## Conclusion

In conclusion, this study revealed that the buffer capacity and calcium concentration of the water used for water kefir fermentation had an impact on the pH and the water kefir grain growth during fermentation. Thus, higher buffer and calcium concentrations of the water used for fermentation increased the water kefir grain growth. Furthermore, the water buffer capacity impacted the microbial communities and their metabolite production during water kefir fermentation. All these data will contribute to the development and upscaling of a stable water kefir production process.

## Data Availability Statement

The datasets generated for this study are available on request to the corresponding author.

## Author Contributions

DL and LD designed the study, performed the experiments, acquired the experimental data except for the culture-dependent microbial species diversity analyses, interpreted the data, and performed the statistical analyses. MA and PV performed the culture-dependent microbial species diversity analyses. DL and LD wrote the manuscript in consultation with PV. All authors provided critical revisions and approved the final version of the manuscript.

## Conflict of Interest

The authors declare that the research was conducted in the absence of any commercial or financial relationships that could be construed as a potential conflict of interest.

## Footnotes

1 http://ndb.nal.usda.gov/
