# Supplementary material for: The Buffer Capacity and Calcium Concentration of Water Influence the Microbial Species Diversity, Grain Growth, and Metabolite Production During Water Kefir Fermentation
Source: Front Microbiol. 2019 Dec 13;10:2876. doi: 10.3389/fmicb.2019.02876 (PMC6923659; doi:10.3389/fmicb.2019.02876)
Supplement: Supplementary file 1 [file Table_1.DOCX]

Supplementary Material

**Table S1.** Characteristics of eight water kefir fermentation series differing in the buffer capacity and calcium concentration of the water used for fermentation at the end of backslopping step 1 [control fermentation with tap water (TAP); fermentations with different buffer capacity and concomitant increasing calcium concentrations (0B0Ca and 0B1Ca; 1B0Ca, 1B1Ca, and 1B4Ca; and 2B1Ca and 2B4Ca); and fermentations with different calcium concentrations and concomitant increasing buffer capacity (0B0Ca and 1B0Ca; 0B1Ca, 1B1Ca, and 2B1Ca; and 1B4Ca and 2B4Ca]. 0B0Ca, no HCO_3_^-^ and Ca^2+^; 0B1Ca, no HCO_3_^-^ and 50 mg l of Ca^2+^; 1B0Ca, 313 mg l^-1^ of HCO_3_^-^ and no Ca^2+^; 1B1Ca, 313 mg l^-1^ of HCO_3_^-^ and 50 mg l^-1^ of Ca^2+^; 1B4Ca, 313 mg l^-1^ of HCO_3_^-^ and 200 mg l^-1^ of Ca^2+^; 2B1Ca, 626 mg l^-1^ of HCO_3_^-^ and 50 mg l^-1^ of Ca^2+^; and 2B4Ca, 626 mg l^-1^ of HCO_3_^-^ and 200 mg l^-1^ of Ca^2+^. Significant differences between the series are indicated with different superscripts (a, b, c, d, and e).

| Characteristic | TAP | 0B0Ca | 0B1Ca | 1B0Ca | 1B1Ca | 1B4Ca | 2B1Ca | 2B4Ca |
| --- | --- | --- | --- | --- | --- | --- | --- | --- |
| Water kefir grain growth (%) | 58.9 ± 1.8 | 57.5 ± 0.3 | 58.0 ± 1.5 | 58.6 ± 1.0 | 60.0 ± 1.0 | 58.1 ± 0.8 | 59.0 ± 1.4 | 58.5 ± 1.5 |
| pH | 3.50 ± 0.04^bc^ | 3.29 ± 0.06 ^e^ | 3.33 ± 0.02 ^e^ | 3.53 ± 0.05 ^abc^ | 3.45 ± 0.05 ^cd^ | 3.41 ± 0.06 ^d^ | 3.59 ± 0.06 ^a^ | 3.56 ± 0.02 ^ab^ |
| Sucrose (g l^-1^) | 1.4 ± 0.1 | 1.5 ± 0.1 | 1.5 ± 0.2 | 1.5 ± 0.1 | 1.5 ± 0.1 | 1.5 ± 0.0 | 1.4 ± 0.1 | 1.5 ± 0.0 |
| Glucose (g l^-1^) | 0.3 ± 0.3 | 0.0 ± 0.1 | 0.2 ± 0.3 | 0.3 ± 0.3 | 0.0 ± 0.0 | 0.0 ± 0.0 | 0.3 ± 0.3 | 0.3 ± 0.2 |
| Fructose (g l^-1^) | 9.1 ± 2.7 | 3.8 ± 1.3 | 6.5 ± 4.9 | 7.3 ± 5.5 | 5.6 ± 1.4 | 5.8 ± 0.7 | 8.7 ± 3.6 | 8.6 ± 2.6 |
| Total residual carbohydrates (g l^-1^) | 10.9 ± 3.0 | 5.3 ± 1.2 | 8.2 ± 5.0 | 9.1 ± 5.8 | 7.1 ± 1.5 | 7.2 ± 0.6 | 10.5 ± 3.8 | 10.4 ± 2.9 |
| Ethanol (g l^-1^) | 16.4 ± 1.4 | 19.4 ± 0.3 | 17.8 ± 2.1 | 16.9 ± 2.3 | 18.4 ± 1.2 | 18.0 ± 0.5 | 16.5 ± 1.0 | 16.9 ± 1.3 |
| Lactic acid (g l^-1^) | 2.61 ± 0.25 | 2.68 ± 0.05 | 2.49 ± 0.36 | 2.60 ± 0.27 | 2.76 ± 0.08 | 2.79 ± 0.07 | 2.67 ± 0.25 | 2.75 ± 0.11 |
| Acetic acid (g l^-1^) | 1.08 ± 0.07^ab^ | 1.00 ± 0.03^b^ | 1.00 ± 0.11^b^ | 1.07 ± 0.08^ab^ | 1.16 ± 0.04^a^ | 1.18 ± 0.09^a^ | 1.14 ± 0.04^a^ | 1.19 ± 0.04^a^ |
| Glycerol (g l^-1^) | 1.90 ± 0.05 | 1.98 ± 0.09 | 1.90 ± 0.14 | 1.87 ± 00.16 | 1.95 ± 0.06 | 1.97 ± 0.10 | 1.89 ± 0.08 | 1.98 ± 0.10 |
| Mannitol (g l^-1^) | 0.81 ± 0.04^ab^ | 0.67 ± 0.09^cd^ | 0.65 ± 0.08^d^ | 0.71 ± 0.08^cd^ | 0.73 ± 0.03^bcd^ | 0.76 ± 0.05^abc^ | 0.82 ± 0.02^ab^ | 0.85 ± 0.06^a^ |
| Glycerol/ethanol (mmol/mol) | 58 ± 4 | 51 ± 3 | 54 ± 3 | 56 ± 3 | 53 ± 2 | 55 ± 2 | 57 ± 3 | 59 ± 4 |
| Lactic acid/ethanol (mmol/mol) | 81 ± 3^ab^ | 71 ± 2^c^ | 72 ± 3^c^ | 79 ± 3^ab^ | 77 ± 3^b^ | 79 ± 3^ab^ | 83 ± 3^a^ | 83 ± 3^a^ |
| Acetic acid/ethanol (mmol/mol) | 51 ± 3^ab^ | 40 ± 1^d^ | 43 ± 2^cd^ | 49 ± 4^ab^ | 48 ± 5^bc^ | 50 ± 5^ab^ | 53 ± 2^ab^ | 54 ± 2^a^ |
| Acetic acid/lactic acid (mol/mol) | 0.62 ± 0.02 | 0.56 ± 0.03 | 0.61 ± 0.03 | 0.62 ± 0.03 | 0.63 ± 0.04 | 0.63 ± 0.04 | 0.64 ± 0.04 | 0.65 ± 0.01 |
| D-lactic acid (% of total) | 45.0 ± 1.0 | 45.7 ± 1.7 | 45.1 ± 0.7 | 44.6 ± 0.5 | 45.0 ± 0.4 | 45.0 ± 1.4 | 45.6 ± 1.5 | 45.2 ± 0.7 |
| Carbon recovery (%) | 100.2 ± 0.8 | 100.9 ± 0.3 | 100.1 ± 1.1 | 99.6 ± 0.3 | 101.6 ± 1.2 | 100.5 ± 0.2 | 100.2 ± 1.0 | 100.9 ± 0.2 |
| 2-Methyl-1-propanol (mg l^-1^) | 8.4 ± 0.7 | 10.4 ± 0.6 | 9.7 ± 1.2 | 9.1 ± 0.5 | 9.9 ± 0.9 | 9.7 ± 0.7 | 9.4 ± 0.4 | 9.3 ± 0.5 |
| Isoamyl alcohol (mg l^-1^) | 41.3 ± 2.9 | 49.5 ± 1.2 | 45.3 ± 4.0 | 44.0 ± 2.1 | 46.7 ± 3.1 | 45.9 ± 1.5 | 45.1 ± 1.4 | 44.6 ± 2.9 |
| Ethyl acetate (mg l^-1^) | 9.9 ± 1.2 | 11.9 ± 0.8 | 11.6 ± 2.0 | 10.1 ± 1.2 | 12.5 ± 1.9 | 12.2 ± 0.7 | 11.5 ± 0.4 | 13.4 ± 2.0 |
| Isoamyl acetate (mg l^-1^) | 0.10 ± 0.01 | 0.14 ± 0.01 | 0.12 ± 0.03 | 0.12 ± 0.03 | 0.13 ± 0.02 | 0.12 ± 0.01 | 0.11 ± 0.01 | 0.11 ± 0.01 |
| Ethyl hexanoate (mg l^-1^) | 0.21 ± 0.01 | 0.27 ± 0.03 | 0.25 ± 0.04 | 0.28 ± 0.07 | 0.29 ± 0.04 | 0.26 ± 0.07 | 0.26 ± 0.01 | 0.25 ± 0.04 |
| Ethyl octanoate (mg l^-1^) | 0.25 ± 0.02 | 0.41 ± 0.15 | 0.31 ± 0.04 | 0.30 ± 0.04 | 0.30 ± 0.04 | 0.33 ± 0.06 | 0.27 ± 0.02 | 0.26 ± 0.03 |

**Table S2.** The pH of eight water kefir fermentation series differing in the buffer capacity and calcium concentration of the water used for fermentation at the end of backslopping steps 1-8 [control fermentation with tap water (TAP); fermentations with different buffer capacity and concomitant increasing calcium concentrations (0B0Ca and 0B1Ca; 1B0Ca, 1B1Ca, and 1B4Ca; and 2B1Ca and 2B4Ca); and fermentations with different calcium concentrations and concomitant increasing buffer capacity (0B0Ca and 1B0Ca; 0B1Ca, 1B1Ca, and 2B1Ca; and 1B4Ca and 2B4Ca]. 0B0Ca, no HCO_3_^-^ and Ca^2+^; 0B1Ca, no HCO_3_^-^ and 50 mg l of Ca^2+^; 1B0Ca, 313 mg l^-1^ of HCO_3_^-^ and no Ca^2+^; 1B1Ca, 313 mg l^-1^ of HCO_3_^-^ and 50 mg l^-1^ of Ca^2+^; 1B4Ca, 313 mg l^-1^ of HCO_3_^-^ and 200 mg l^-1^ of Ca^2+^; 2B1Ca, 626 mg l^-1^ of HCO_3_^-^ and 50 mg l^-1^ of Ca^2+^; and 2B4Ca, 626 mg l^-1^ of HCO_3_^-^ and 200 mg l^-1^ of Ca^2+^. Significant differences between the series are indicated with different superscripts (a, b, c, d, and e).

| Backslopping step | TAP | 0B0Ca | 0B1Ca | 1B0Ca | 1B1Ca | 1B4Ca | 2B1Ca | 2B4Ca |
| --- | --- | --- | --- | --- | --- | --- | --- | --- |
| 1 | 3.50 ± 0.04 ^bc^ | 3.29 ± 0.06 ^e^ | 3.33 ± 0.02 ^e^ | 3.53 ± 0.05 ^abc^ | 3.45 ± 0.05 ^cd^ | 3.41 ± 0.06 ^d^ | 3.59 ± 0.06 ^a^ | 3.56 ± 0.02 ^ab^ |
| 2 | 3.45 ± 0.03 ^cd^ | 3.30 ± 0.06 ^e^ | 3.25 ± 0.01 ^e^ | 3.52 ± 0.07 ^bc^ | 3.45 ± 0.02 ^cd^ | 3.41 ± 0.02 ^d^ | 3.60 ± 0.03 ^a^ | 3.56 ± 0.07 ^ab^ |
| 3 | 3.43 ± 0.05 ^c^ | 3.26 ± 0.06 ^d^ | 3.23 ± 0.0 ^d^ | 3.46 ± 0.01 ^c^ | 3.43 ± 0.05 ^c^ | 3.42 ± 0.03 ^c^ | 3.64 ± 0.01 ^a^ | 3.54 ± 0.07 ^b^ |
| 4 | 3.44 ± 0.02 ^b^ | 3.24 ± 0.03 ^e^ | 3.20 ± 0.02 ^e^ | 3.43 ± 0.03 ^bc^ | 3.38 ± 0.03 ^d^ | 3.38 ± 0.03 ^cd^ | 3.61 ± 0.04 ^a^ | 3.46 ± 0.02 ^b^ |
| 5 | 3.35 ± 0.01 ^b^ | 3.17 ± 0.03 ^c^ | 3.17 ± 0.01 ^c^ | 3.36 ± 0.04 ^b^ | 3.36 ± 0.04 ^b^ | 3.33 ± 0.04 ^b^ | 3.52 ± 0.06 ^a^ | 3.47 ± 0.04 ^a^ |
| 6 | 3.42 ± 0.02 ^cd^ | 3.18 ± 0.01 ^e^ | 3.20 ± 0.04 ^e^ | 3.42 ± 0.01 ^bc^ | 3.39 ± 0.02 ^cd^ | 3.37 ± 0.03 ^d^ | 3.58 ± 0.05 ^a^ | 3.47 ± 0.02 ^b^ |
| 7 | 3.35 ± 0.03 ^b^ | 3.14 ± 0.06 ^c^ | 3.13 ± 0.02 ^c^ | 3.39 ± 0.06 ^b^ | 3.35 ± 0.04 ^b^ | 3.33 ± 0.09 ^b^ | 3.54 ± 0.02 ^a^ | 3.47 ± 0.02 ^a^ |
| 8 | 3.45 ± 0.01 ^bc^ | 3.17 ± 0.01 ^e^ | 3.14 ± 0.02 ^e^ | 3.41 ± 0.04 ^c^ | 3.43 ± 0.10 ^c^ | 3.32 ± 0.03 ^d^ | 3.60 ± 0.01 ^a^ | 3.52 ± 0.02 ^b^ |

**Table S3.** The water kefir grain growth of eight water kefir fermentation series differing in the buffer capacity and calcium concentration of the water used for fermentation [control fermentation with tap water (TAP); fermentations with different buffer capacity and concomitant increasing calcium concentrations (0B0Ca and 0B1Ca; 1B0Ca, 1B1Ca, and 1B4Ca; and 2B1Ca and 2B4Ca); and fermentations with different calcium concentrations and concomitant increasing buffer capacity (0B0Ca and 1B0Ca; 0B1Ca, 1B1Ca, and 2B1Ca; and 1B4Ca and 2B4Ca]. 0B0Ca, no HCO_3_^-^ and Ca^2+^; 0B1Ca, no HCO_3_^-^ and 50 mg l of Ca^2+^; 1B0Ca, 313 mg l^-1^ of HCO_3_^-^ and no Ca^2+^; 1B1Ca, 313 mg l^-1^ of HCO_3_^-^ and 50 mg l^-1^ of Ca^2+^; 1B4Ca, 313 mg l^-1^ of HCO_3_^-^ and 200 mg l^-1^ of Ca^2+^; 2B1Ca, 626 mg l^-1^ of HCO_3_^-^ and 50 mg l^-1^ of Ca^2+^; and 2B4Ca, 626 mg l^-1^ of HCO_3_^-^ and 200 mg l^-1^ of Ca^2+^. Significant differences between the series are indicated with different superscripts (a, b, c, d, e, and f).

| Backslopping step | TAP | 0B0Ca | 0B1Ca | 1B0Ca | 1B1Ca | 1B4Ca | 2B1Ca | 2B4Ca |
| --- | --- | --- | --- | --- | --- | --- | --- | --- |
| 1 | 58.9 ± 1.8 | 57.5 ± 0.3 | 58.0 ± 1.5 | 58.6 ± 1.0 | 60.0 ± 1.0 | 58.1 ± 0.8 | 59.0 ± 1.4 | 58.5 ± 1.5 |
| 2 | 51.7 ± 1.5 ^ab^ | 38.8 ± 3.8 ^d^ | 44.1 ± 1.3 ^c^ | 51.3 ± 0.1 ^ab^ | 51.0 ± 0.6 ^b^ | 52.6 ± 1.1 ^ab^ | 52.7 ± 1.7 ^ab^ | 54.6 ± 3.2 ^a^ |
| 3 | 50.1 ± 1.3 ^bc^ | 20.9 ± 6.7 ^e^ | 35.9 ± 2.7 ^d^ | 48.1 ± 2.5 ^c^ | 49.3 ± 1.0 ^bc^ | 51.9 ± 1.0 ^ac^ | 55.5 ± 2.1 ^a^ | 54.0 ± 1.4 ^ab^ |
| 4 | 49.5 ± 0.9 ^c^ | 11.4 ± 4.9 ^f^ | 25.3 ± 2.7 ^e^ | 43.6 ± 2.0 ^d^ | 48.7 ± 1.4 ^c^ | 52.5 ± 2.7 ^bc^ | 54.6 ± 1.5 ^ab^ | 58.0 ± 2.1 ^a^ |
| 5 | 49.3 ± 2.0 ^b^ | 6.8 ± 1.5 ^f^ | 18.5 ± 3.9 ^e^ | 39.5 ± 2.6 ^d^ | 43.6 ± 1.0 ^c^ | 51.7 ± 1.1 ^b^ | 52.8 ± 1.9 ^b^ | 57.3 ± 2.6 ^a^ |
| 6 | 48.5 ± 1.1 ^c^ | 5.1 ± 1.0 ^g^ | 15.9 ± 3.4 ^f^ | 34.3 ± 2.8 ^e^ | 42.1 ± 1.7 ^d^ | 49.8 ± 1.0 ^bc^ | 53.0 ± 2.2 ^ab^ | 55.5 ± 2.2 ^a^ |
| 7 | 49.9 ± 1.6 ^bc^ | 2.2 ± 0.4 ^g^ | 8.0 ± 1.6 ^f^ | 29.0 ± 2.8 ^e^ | 35.8 ± 3.3 ^d^ | 48.5 ± 2.6 ^c^ | 54.0 ± 1.1 ^a^ | 53.1 ± 2.3 ^ab^ |
| 8 | 47.9 ± 0.8 ^a^ | 2.7 ± 0.5 ^d^ | 5.4 ± 0.6 ^d^ | 17.5 ± 1.8 ^c^ | 31.2 ± 8.7 ^b^ | 47.2 ± 0.5 ^a^ | 50.9 ± 3.0 ^a^ | 52.0 ± 2.3 ^a^ |
